# Reproducibility in the unfolding process of protein induced by an external electric field†

**DOI:** 10.1039/d0sc06008a

**Published:** 2020-12-26

**Authors:** Anna Sinelnikova, Thomas Mandl, Christofer Östlin, Oscar Grånäs, Maxim N. Brodmerkel, Erik G. Marklund, Carl Caleman

**Affiliations:** Department of Physics and Astronomy, Uppsala University Box 516 SE-751 20 Uppsala Sweden carl.caleman@physics.uu.se; University of Applied Sciences Technikum Wien Höchstädtplatz 6 A-1200 Wien Austria; Department of Chemistry – BMC, Uppsala University Box 576 SE-751 23 Uppsala Sweden erik.marklund@kemi.uu.se; Center for Free-Electron Laser Science, DESY Notkestrasse 85 DE-22607 Hamburg Germany

## Abstract

The dynamics of proteins are crucial for their function. However, commonly used techniques for studying protein structures are limited in monitoring time-resolved dynamics at high resolution. Combining electric fields with existing techniques to study gas-phase proteins, such as single particle imaging using free-electron lasers and gas-phase small angle X-ray scattering, has the potential to open up a new era in time-resolved studies of gas-phase protein dynamics. Using molecular dynamics simulations, we identify well-defined unfolding pathways of a protein, induced by experimentally achievable external electric fields. Our simulations show that strong electric fields in conjunction with short-pulsed X-ray sources such as free-electron lasers can be a new path for imaging dynamics of gas-phase proteins at high spatial and temporal resolution.

## Abstract 2

Controlled unfolding of proteins can reveal structural properties and give insights of the proteins' dynamics. We show the feasibility of unfolding proteins in the gas phase using electric fields, with a well-defined path at high field strengths.

## Graphical abstract

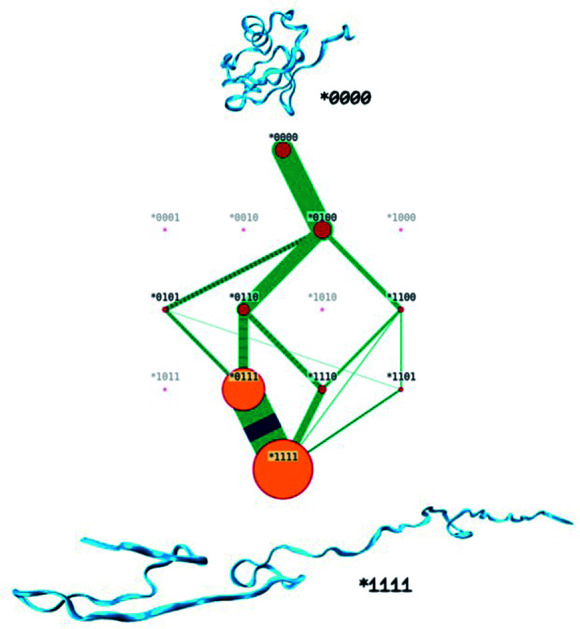

## 1 Introduction

Proteins are innately dynamic, their motions ranging from the vibrations of atoms to large rearrangements on the tertiary or quaternary level, spanning timescales from femtoseconds to milliseconds and beyond.^1^ Protein dynamics on all levels have importance for protein function,^2,3^ and new functions may also evolve from pre-existing conformational sub-states.^4^ Understanding protein dynamics is consequently central for understanding the functions and evolution of proteins, and studying static structures might be insufficient to this end.^5,6^ A protein's mechanical properties, underpinning its dynamics, can be investigated by inducing stress on the protein and monitoring the response, which in turn can be affected by interactions with ligands, such as therapeutic drugs or other macromolecules.^7,8^ Unfolding studies are carried out for this purpose, using, for example, atomic-force microscopy^9^ or optical tweezers^10^ to obtain information about the strengths of domains and (un)folding pathways, or elevated temperatures to assess stability.^7^ Further, there are large numbers of simulation studies of the effect electric fields have on the structures of biological molecules,^11^ primarily in the liquid phase,^12–15^ but also in gas phase.^16^ Native Mass Spectrometry (MS), in which intact protein complexes are transferred to the gas phase with maintained non-covalent interactions, has also proven useful in this respect,^17^ in part due to its ability to probe specific states and its wide repertoire of methods for manipulating the proteins as they pass through the mass spectrometer. Together with Ion Mobility spectrometry (IM), MS can probe how the overall structure changes as the protein takes up increasing amounts of energy,^18–20^ uncovering the stabilizing effects of different ligands^8^ or differences between closely related proteins.^21^ Despite its many advantages, IM-MS provides structural information that is relatively scarce. This is unfortunate, since the relation between the atomic structure and its dynamics, and how a protein functions *in vivo*, links physics and chemistry to biology and medicine. Consequently, better structural resolution in dynamics studies would ultimately allow for more insights about the protein and its role in cellular function.

The aerosolization techniques used to deliver intact protein complexes in IM-MS lend themselves to another emerging technique to reveal the structures of gas phase macromolecules,^24^ Single Particle Imaging (SPI). SPI employs powerful X-ray Free-Electron Lasers (XFELs), exposing individual macromolecules to ultra-short and -bright X-ray pulses that scatter to generate diffraction patterns from which the structures can be inferred.^25^ In principle, this enables SPI to harness many of the manipulations offered by MS while providing detailed structural information about the protein under study. Although nanometer resolution of a virus particle has been presented using SPI,^26^ providing proof of concept, atomic resolution of single biomolecules has so far not been achieved. To reach the desirable atomic resolution the technique has to be improved, and IM-MS might provide one step forward. Proof-of-principle experiments that utilize MS for sample delivery show that Small Angle X-ray Scattering (SAXS) can provide structural information about proteins in the gas phase.^27^ When combined with our recently proposed field-orientation of gas-phase proteins,^28^ MS becomes an even more promising addition to X-ray diffraction of proteins in the gas phase. Using Molecular Dynamics (MD) simulations we showed that applying an electric field with the right field strength would orient a protein without unfolding it, with clear benefits for the reconstruction of the structure from the diffraction patterns, which is currently pursued experimentally within the MS-SPIDOC Horizon 2020 project (http://www.ms-spidoc.eu). If on the other hand the field strength is too high, the electrostatic forces become destructive for the structure and the protein unfolds. An undesired phenomenon at fist glance, but one which could be exploited to trigger protein unfolding just prior to X-rays exposure, enabling probing of the protein structure as it unfolds. If the unfolding is also reproducible, SPI or SAXS would be even more informative, enabling time-resolved imaging of the unfolding pathway.

In this study we address two main questions through simulations of the globular protein ubiquitin: (i) how reproducible is the protein unfolding caused by an external electric field? (ii) Can we identify well defined unfolding pathways? We further discuss the possibilities of detecting this unfolding using different experimental approaches. Building on earlier methodology, we employ MD simulations^28^ to investigate the molecular response of the protein to the electric field, and quantum mechanics for the electronic response.^29^

## 2 Method

### 2.1 Molecular dynamics simulations

As in our earlier studies,^28,30^ we used the MD package GROMACS^31^ in combination with the OPLS/AA^32,33^ force field parameters. We chose the protein ubiquitin (1UBQ^34^) for this study. Ubiquitin has been extensively simulated in the gas phase using several force fields (OPLS/AA, AMBER03 (ref. 35) and GROMOS96 53a6 (ref. 36)), and was shown to be more structurally stable than similar sized proteins in vacuum.^37^ Thermally^38^ and mechanically^22^ induced unfolding of ubiquitin has been extensively studied, and its unfolding pathways are well known.

We followed a similar simulation protocol as described in Marklund *et al.*^28^ In short we did the following: the dipole of the initial structure was aligned with the electric field, assuming a pre-oriented sample. From this starting configuration, 100 independent simulations were performed at each field strength. Each simulation was 50 ps long, and the Leap-Frog integration scheme with a 0.5 fs time-step was used. The protein was exposed to a constant external electric field at field strengths between 3 × 10^4^ kV cm^−1^ and 11 × 10^4^ kV cm^−1^. 3 × 10^4^ kV cm^−1^, is the strength where we have seen that unfolding starts to manifest itself^28^ for the protein considered here. For less complex organic molecules, polyalanines, simulations have shown that electric field affects the structure at even lower strengths,^16^ but for ubiquitin and the timescales we are considering here, 3 × 10^4^ kV cm^−1^ seems to be the limit. The highest value we simulated, 11 × 10^4^ kV cm^−1^ is considered an extreme value. Four different field strengths were then picked up to focus on, based on difference in the unfolding dynamics: 3 × 10^4^ kV cm^−1^, 5 × 10^4^ kV cm^−1^, 7 × 10^4^ kV cm^−1^ and 11 × 10^4^ kV cm^−1^.

We averaged the data over 100 independent runs and plotted the mean value together with one-sigma deviation of the average, defined as1
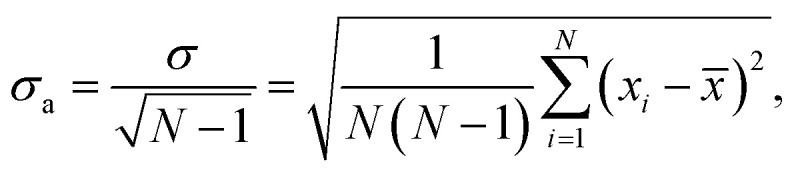
where *σ* is the standard deviation.

### 2.2 Analyzing unfolding pathways

For each value of the electric field strength we simulated 100 statistically independent runs. The initial configuration was extracted from the simulation trajectories as described in Marklund *et al.* and stayed the same for all the runs we describe in this paper.

**Fig. 1:**
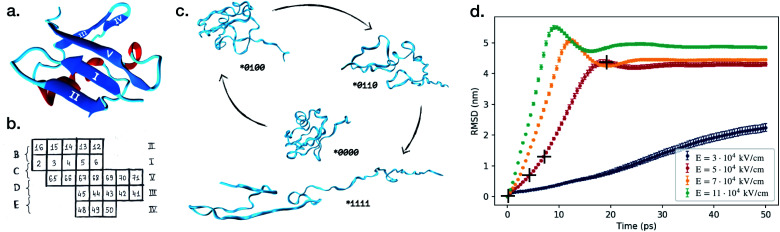
(a) Native protein conformation of ubiquitin with a schematic illustration of its secondary structure.^22^ (b) Five β-strands are labeled with Roman numerals and the couplings between them are marked with B–D letters. We have used a binary notation to describe if the β-strand couplings B–E are broken or not, where 0 means connected and 1 means broken. For example, (*0100) denotes that only the C-coupling is broken. Letter A is reserved for the α-helix. (c) The process of unfolding of ubiquitin over time in the electric field *E* = 5 × 10^4^ kV cm^−1^. Snapshots were taken at 0 ps, 4.47 ps, 7.95 ps and 19.35 ps after turning on the electric field. (d) Average root mean square displacement, RMSD, and standard deviation of the average value for the simulations in the four different electric fields. Each line represents a set of 100 independent simulations, and the RMSD is calculated at each time step comparing the 100 structures at this specific time in the simulations. A similar trend can be observed for the average radius of gyration, depicted in Fig. S1 in the ESI.† The crosses mark the time points the structures in (c) were taken from. We used VMD software for the visualisation.^23^

**Fig. 2:**
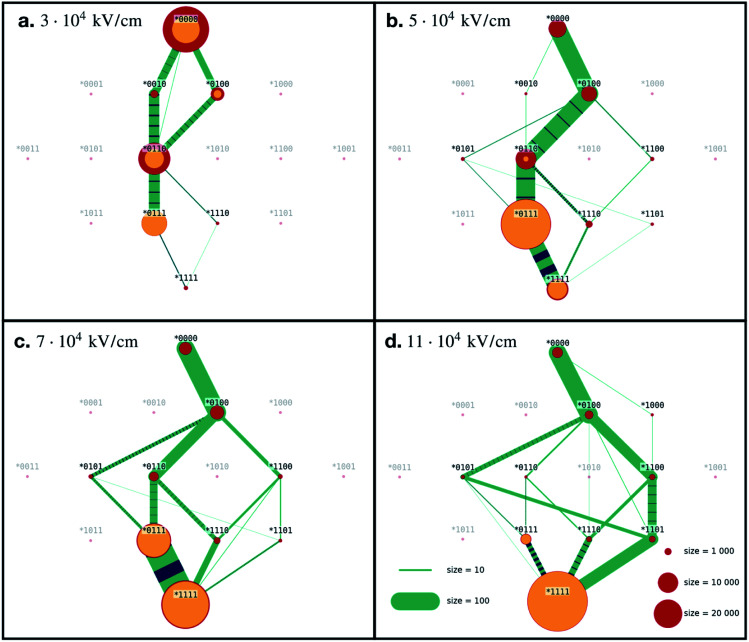
Transition maps for various values of the electric field. Each point represents one state *BCDE, where each of four structures can be either folded (the corresponding bit is 0) or unfolded (the corresponding bit is 1). The color of the points indicates if it is a final state (yellow) or a transition state (red). The thickness of the lines indicates the frequency of the transition between two states. Green color means transition from a folded state (0) to an unfolded state (1), and blue lines the opposite. Striped lines demonstrate transitions in both directions.

Ubiquitin in the native conformation consists of one α-helix and five β-strands like shown in Fig. 1. Following the notation by Irbäck *et al.*,^22^ we defined the coupling between the β-strands forming the β-sheet in the following way (see Fig. 1), (B): I and II connected, (C): I and V connected, (D): III and V connected and (E): III and IV connected. We further defined the α-helix as (A). We used binary representation “folded/unfolded” for plotting the transition map presented in Fig. 2. To every secondary structure A, B, C, D, E we assigned a bit. If the structure is unfolded, the value of the bit is 1, otherwise the value is 0. Thus, we can characterize ubiquitin by the 5 bit state ABCDE. The native conformation corresponds to 00000 whilst the completely unfolded state is denoted as 11111. Next, we had to introduce some quantitative criteria for detecting unfolding in each of the five secondary structures. For β-strands couples B, C, D and E we measured the distance between centre of masses of each strand within the couple. For instance, for the strand couple which we call B, we calculated the distance between centre of mass of strand I and centres of mass of strand II. Hence, we considered the strands disconnected if this distance exceeded 0.7 nm. This value was chosen to empirically provide the most clear diagrams, and does not have any physical justification other than that increasing distances eventually leads to unfolding. A lower value would give less clear pathways in our diagrams, but the main conclusion we draw would stay the same. Setting the threshold to a fixed value gives us a relative quantity to compare different ways of the unfolding of the β-sheets. For α-helix A we unavoidably had to make some other criteria. We calculated the end-to-end distance for different threshold values, which only introduced noise to the transition maps due to fast osculations of that value. Therefore, we decided to excluded A from the scope and considered 4 bit states for couples of β-strands *BCDE. A transition map based on our simulation trajectories was created, depicted in Fig. 2 as a graph with 2^4^ = 16 vertices. The system was probed every d*t* = 0.05 ps to observe its state, *i.e.* each link in Fig. 2 has a meaning of probability of transition in next 0.05 ps. If the system remained in its state, we increased the size of the corresponding vertex. If a transition happened, we connected the corresponding vertices with a link. The map represented in Fig. 2 should be understood as a representation of the Markovian transition matrix: one cannot see the full path of any simulation, but only the transitions between two certain states (or between the same states – remaining in the position). In the map, frequently occurring transitions are indicated by thicker lines. Transition lines from a folded state (0) to an unfolded state (1) are depicted green, while transitions in the opposite direction are colored blue. Blue-green striped lines mean that the transition goes in both ways and the thickness of the stripes shows the frequency of the corresponding direction.

The source of the diagram is always the state *0000 native configuration, but the final state, the sink, can be any state, since not all of the β-strands necessarily unfold. Sinks are colored yellow, red indicates a temporary state. The size of the dots represent how long the system spent in this final state. For visibility, the width of the transition lines scales linearly while the states size scales as the area of the dot.

### 2.3 Estimation of field-induced electron redistribution and field emission

Using the Siesta 4.1 software,^39^ we first thermalized the protein using Born–Oppenheimer MD. We used the Nosé thermostat to reach an ionic temperature of 300 K. For the thermalization procedure we used a double-Z basis set with a (single-Z) polarization orbital per atom. The confining potential was kept to 0.001 Ry in order to allow for sufficiently long-ranged radial basis functions, whereas the numerical integration grid was determined by a 200 Ry cut-off. The exchange-correlation was treated according to the van der Waals function by Vydrov and van Voorhis.^40^ The initial thermalization procedure was set to 2 ps, 5 trajectories were simulated. The simulated trajectories were then put in an external field of magnitudes 3 × 10^4^, 5 × 10^4^, 7 × 10^4^ and 11 × 10^4^ kV cm^−1^. For these simulations, the basis set was extended to encompass the changes in the electron distribution with respect to the ground-state. We used a triple-Z basis set with a double set of polarization orbitals. The integration mesh cut-off was increased to 500 Ry in order to get accurate partial charges. We analyzed the redistribution in terms of how partial occupations changed with respect to the ground-state.

In order to benchmark the impact of the explicit treatment of the electron density *vs.* the classical description of atoms in MD simulations, we calculated the relative change in the dipole of an unfolded and folded Trp-cage protein. As expected, the changes in the dipole was larger in an unfolded protein than in its folded state. We applied an electric field in the direction opposite to the proteins intrinsic dipole, mimicking the situation where the protein is oriented. A table of the induced dipoles can be seen in the ESI (Table S1†). The charge distribution polarized in response to the applied field. The field-induced changes relative to the difference between folded and unfolded proteins were relatively small until roughly 7 × 10^4^ kV cm^−1^, where the unfolded protein reached a state of internal dielectric breakdown. Bear in mind that the unfolded protein is almost twice as long as its folded counterpart (37 Å *vs.* 20 Å), making the difference in field-induced potential significantly larger between the end points.

### 2.4 Analyzing diffraction patterns

The flat virtual detector mimicked an AGIPD with 1024 × 1024 square pixels, each with a side length of 200 μm. It was placed 25 cm downstream from the sample with respect to the X-ray beam, which allows for scattering up to a resolution of 3.5 Å using photon energies of 8 keV (*λ* = 1.55 Å). The pulse intensity was 10^12^ instantaneously arriving photons, uniformly distributed over a 100 nm in diameter focal spot.

Diffraction patterns from a single frame at time *t* from any given simulation trajectory were calculated using an in-house code developed by Martin^41^ and used in our previous work.^42,43^ It evaluates the pixel intensities *I*(**q**,*t*) forming the pattern as2
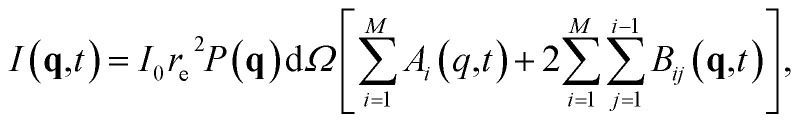
where **q** is the scattering vector (*q* being its magnitude), *I*_0_ is the incident intensity, *r*_e_ is the classical electron radius, *P*(**q**) is a polarization term, d*Ω* is the solid-angle of the pixel and *M* is the total number of atoms in the trajectory frame. *A*_*i*_(*q*,*t*) and *B*_*ij*_(**q**,*t*) give the intensity contributions from individual atoms and the cross-correlation between them, respectively. They are calculated from tabulated atomic X-ray form factors *f*_*i*_(*q*,*t*) for the different elements as

3 *A*_*i*_(*q*,*t*) = |*f*_*i*_(*q*,*t*)|^2^,

4 *B*_*ij*_(**q**,*t*) = *f*_*i*_(*q*,*t*)*f*_*j*_(*q*,*t*)cos{2π**q**(**R**_*i*_(*t*) − **R**_*j*_(*t*))}.

Here, the position of atom *i* is represented by the vector **R**_*i*_ and the form factors used are spherically symmetric.

Individually calculated patterns represent converged averages from identical particles, meaning that no damage processes or noise contributions are considered. However, since patterns from the same time point of 100 independent MD runs were combined in our analysis, noise stemming from sample heterogeneity is accounted for. Time points chosen were separated 5 ps apart, starting with the initial (*t* = 0 ps) and ending with the final frame (*t* = 50 ps), for a total of 11 frames.

### 2.5 Estimating alpha carbon distances

We calculated the distances between all pairs of α-carbons for all saved time steps and averaged those distances over all runs of a certain field strength, giving us mean distances as a function of field exposure time. In a further step, we calculated the time average for two phases, 0–10 ps and 20–50 ps, and plotted them as ‘distance maps’, displayed in Fig. S8 in the ESI.† A pixel corresponds to a pair of α-carbons, its color represents the average distance. With dark regions indicating closeness of corresponding residues, the distance maps reveal structures of the protein such as β-strands. Comparing distance maps at different times allows the observation of the breaking of structures A–E. The lower part of the distance maps is intentionally left empty (dark), as it would not contain additional information due to the symmetry of the distance between pairs of atoms.

## 3 Results

We start with investigating how the structure of ubiquitin evolves when exposed to electric fields of different strengths. For four different field strengths, we ran 100 independent short simulations each, from which we could derive the protein's characteristic behavior upon exposure to the field. Fig. 1d shows the time-dependent root-mean square deviation of the atomic positions (RMSD). The figure depicts the RMSD averaged over all independent simulations, and the error bars describe the standard deviation from the average value. The most striking observation is that the error bars are significantly smaller than the mean values of the RMSD, across all field strengths. This already answers the first question we posed: the proteins do unfold in a similar way each time.

We can further identify three trends from Fig. 1: (i) stronger fields create a larger deviation from the original configurations. (ii) Stronger fields increase the unfolding speed, at fields of 5 × 10^4^ kV cm^−1^ the unfolding occurs within 20 ps, whereas it only takes 10 ps in a 11 × 10^4^ kV cm^−1^ field. (iii) The third is the most important and interesting observation: at stronger fields, the standard deviation of the RMSD at each point in time is lower. This means that, when unfolding with the higher fields, the unfolding process of the 100 simulations are more similar to each other than at low fields, *i.e.* it seems like the unfolding in the stronger fields is more reproducible.

The RMSD primarily gives information about how much the protein structure deviates from the original structure, indicating if the protein is folded or not. However, to answer the second question one would need to make a more detailed study and investigate the *pathways* of unfolding. We investigate the breaking of five secondary structures A, B, C, D and E according to the notation (see Fig. 1 or the Method section for definition).

For the lowest field we simulated, *E* = 3 × 10^4^ kV cm^−1^, we see that in some of our simulations the proteins did not completely unfold, and very few reached the final state (*1111). This goes in line with the RMSD (Fig. 1d), where we saw that the weakest field could not provide full unfolding, at least not within the 50 ps that we have simulated the systems. In these simulations, we see a range of final states: connection C broken (*0100), C and D broken (*0110), or C, D and E broken (*0111). The state where only D is broken (*0010) seems to be unstable, because even though the system visited it, the state was never a final state of the process. The same is true for the fully unfolded conformation (*1111).

In Fig. 2 we present the transition maps for the unfolding pathways for the four different field strengths. For the field *E* = 5 × 10^4^ kV cm^−1^, we can see a well defined unfolding pathway. The first structure to break is C, (*0100), followed by D (*0110), E (*0111) and finally B (*1111). Hence the order of unfolding the β-strands is CDEB. The fully unfolded state (*1111) is less stable than the state where only B remained folded (*0111). We can see a large transition from (*1111) to (*0111), indicated by the striped transition line.

For the *E* = 7 × 10^4^ kV cm^−1^ field, we observe (in contrast to the lower fields) that the fully unfolded state is preferred by the system. We still see the same main pathway as for *E* = 5 × 10^4^ kV cm^−1^, CDEB. However, B can unfold before D with higher probability than in the weaker field, *i.e.* the system reaches the state (*1100) instead of the (*0110) state. We can also notice that E has a small probability to break before D, *i.e.* state (*1101) before state (*1111). If this happens, the unfolding pathway is CEDB, rather than CDEB.

The results for the strongest field we simulated, *E* = 11 × 10^4^ kV cm^−1^, is presented in Fig. 2d. The main difference in the unfolding pathways compared to Fig. 2b, is that here the main pathway seems to be CBDE and not CDEB. Low frequent order of breaking BD (state (*1100) followed by (*1110)) in the previous case now became one of the leading behaviours. As in the case of *E* = 7 × 10^4^ kV cm^−1^, we can see a small probability that E (*0101) breaks before B (*1100), resulting in an order of CEBD.

Looking at all four diagrams, one can notice that C is the first to unfold, but it is almost never folded back. The only case where we have transition from (**1**) to (**0**) is the first diagram, Fig. 2a. Here, C folded back only after D was unfolded first, *i.e.* following the path (*0000), (*0100), (*0110), (*0010).

Two main unfolding pathways can be seen in the simulations. For the strongest fields (*E* = 11 × 10^4^ kV cm^−1^), we see CBED, and for the lower fields (*E* ≤ 7 × 10^4^ kV cm^−1^) CDEB.

To investigate this further, we have studied in detail in which order the two connections B and D break. If B breaks first, we have the order CBED, and if D breaks first, we have the order CDEB. We have analysed the time points for the breaking of B and D at three fields strengths. As we increase the electric field, the probability for B breaking before D increases accordingly, meaning the dominating unfolding pathway changes from CBED to CDBE, as depicted in Fig. S2 in the ESI.†

Exposing gas-phase proteins to the high electric fields that we describe here affect the electronic structure of the molecules and could potentially lead to field emission. We have investigated such a potential impact on the electronic structure by quantum mechanical simulations based on Density-Functional Theory (DFT). As a model system, we chose the synthetic protein Trp-cage, which is small enough to allow for sufficient numerical accuracy to address charge redistribution.

The purpose of this is two fold, (i) we want to investigate the electronic rearrangement and possibility of field emission, and (ii) reassure that the force-field we employ in our MD simulations is accurate under the influence of an electric field. To estimate the impact of the unfolding procedure, we present results for folded and unfolded Trp-cage.

The electron redistribution for the folded Trp-cage induces a displacement of charge that is field-dependent. The maximum changes in charge on any individual atom accumulates on hydrogen atoms close to the edges of the protein. This is a natural effect of the potential induced by the field, in combination with the relatively weak coulomb potential of the nucleus of the hydrogen atom. To evaluate the difference, we use the atomic Mulliken charges,^44^ denoting differences Δ*M*_c_. From Table 1, we see that allowing for charge to relax in the applied electric field, we get substantial contributions for electric fields of 7 × 10^4^ kV cm^−1^ for the unfolded protein. The maximum Mulliken charge differences resides on hydrogen in all cases, indicating that there is a substantial risk of altering the hydrogen bonds in the system when the fields reach 7 × 10^4^ kV cm^−1^ or higher.

**Table 1:** Maximum and minimum differences in Mulliken charges of Trp-cage with and without applied field. Units are in electronic charge *e*

*E* (kV cm^−1^)	Folded		Unfolded	
	Max. (Δ*M*_c_)	Min. (Δ*M*_c_)	Max. (Δ*M*_c_)	Min. (Δ*M*_c_)
3 × 10^4^	0.02	−0.02	0.05	−0.03
5 × 10^4^	0.04	−0.07	0.16	−0.06
7 × 10^4^	0.18	−0.13	0.32	−0.11
11 × 10^4^	0.50	−0.23	0.68	−0.21

Further, we analyze the difference in molecular dipole moment *μ* in the folded and the unfolded states calculated with DFT and with molecular mechanics with respect to field. For field strengths below 5 × 10^4^ kV cm^−1^, the change in dipole moment due to the unfolding procedure dominates. For higher field strengths, the potential increase of electrons on one side surpasses a threshold barrier for relocating charge, and the molecular dipole moment changes dramatically.

As mentioned in the introduction, one potential way of studying the unfolding of protein molecules experimentally is through the emerging technique of single particle X-ray imaging. Ideally, high-resolution and radiation damage-free diffraction patterns can be measured from single molecules using the high-intensity, ultra-short X-ray pulses offered by free-electron lasers. To simulate such data, diffraction patterns were calculated from the MD simulations. Parameters were chosen to reflect those of the recently commissioned SPB/SFX beamline at the European XFEL,^45^ an instrument designed for such experiments.

The calculated diffraction patterns were analyzed in two distinct procedures, each requiring the averaging within a time step to be handled differently. In the first, we aimed to compare how heterogeneity between analogous simulation runs limits the resolution in diffraction space through Fourier Ring Correlation (FRC). By dividing the set of 100 patterns into two equally-sized subsets and averaging the subsets individually, we obtained two separate patterns representative of what an SPI algorithm aims to reconstruct. At higher resolutions – *i.e.* far from the detector center – these patterns will exhibit larger deviations, since smaller real space differences become more influential. To perform FRC, the patterns are partitioned into equal resolution rings, which are then correlated between the patterns individually. The resulting function shows how well-correlated the two data sets are at the different resolutions. It is calculated as5
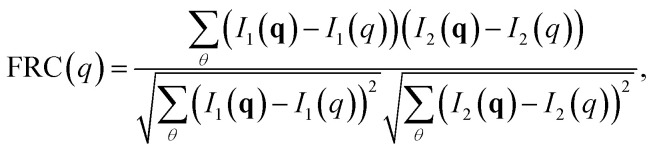
where *I*_*j*_(**q**) are the two average patterns, *I*_*j*_(*q*) are the mean intensities in the resolution rings corresponding to scattering vector length *q*, and the sum over *θ* describes the discretized integration around the ring since **q** = (*q*, *θ*) in polar coordinates. The FRC attains values in the interval [−1, 1], where the two extremes indicate perfect negative and positive linear correlation, respectively. The midpoint, conversely, is the result when two resolution rings are uncorrelated.

**Fig. 3:**
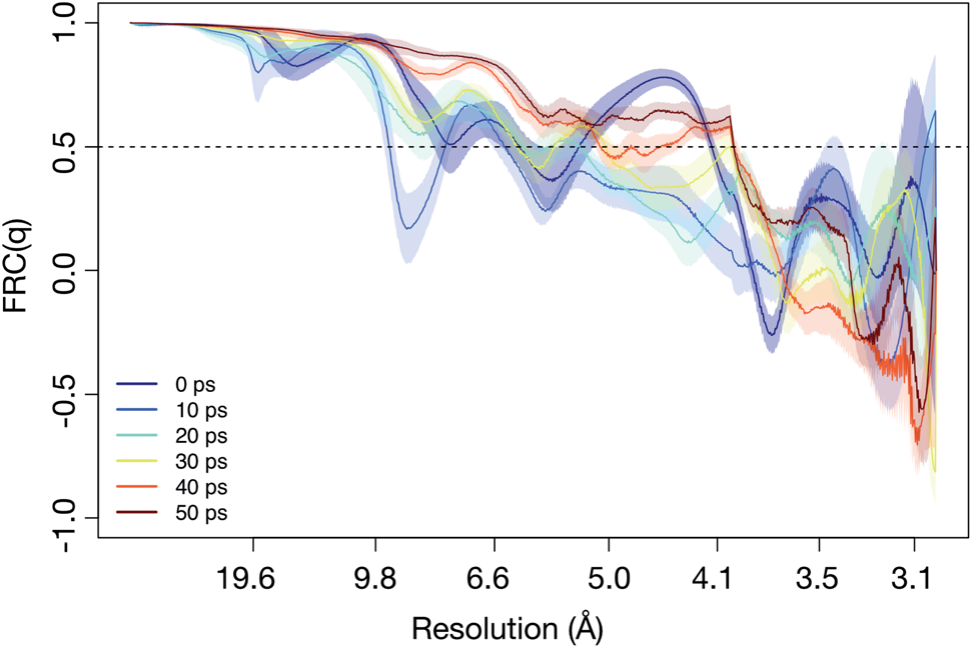
Estimated resolution limit, based on Fourier ring correlation (FRC) for different time points in the simulation. During the unfolding process, the resolution decreases (at 10 ps to 20 ps, compare to Fig. 1d). The simulation depicted utilized an electric field of *E* = 3 × 10^4^ kV cm^−1^, plots for other fields can be found in Fig. S3 in the ESI.†

To determine a theoretical limit to the obtainable resolution in SPI, we used a constant cut-off of FRC(*q*) = 0.5, a threshold that has been used previously.^46^ That is, the largest *q* = *q*_max_, where FRC(*q*) > 0.5 for all *q* < *q*_max_ was considered the momentum transfer up to which structural information remained consistent between the two subsets. Real space resolution is related to the length of the scattering vector as 1/*q* in our calculations, so 1/*q*_max_ was defined as the resolution limit. Note that this number should not be interpreted as the best realistically achievable resolution in an experimental setting, but rather represents an idealized case and allows for comparisons between time points and electric field strengths. We compare the FRC as a function of time for all the simulations, and compare it with the cut-off. Fig. 3 illustrates this for 3 × 10^4^ kV cm^−1^ (for the other field strengths we refer to Fig. S3 in the ESI†). What we find is that for all the field strengths above 3 × 10^4^ kV cm^−1^, the achievable resolution of the unfolding process is around 4 Å for any time point we considered. For 3 × 10^4^ kV cm^−1^, however, the resolution is lower in the earlier part of the unfolding process, but reaches around 4 Å after around 50 ps. This is caused by the two unfolding pathways ((*0000) → (*0010) → (*0110) or (*0000) → (*0100) → (*0110)) (compare to Fig. 2), resulting in a lower resolution in the time frame when the protein occupies these confirmation states. For the higher fields strengths, the unfolding pathways are dominated by one well-defined path.

**Fig. 4:**
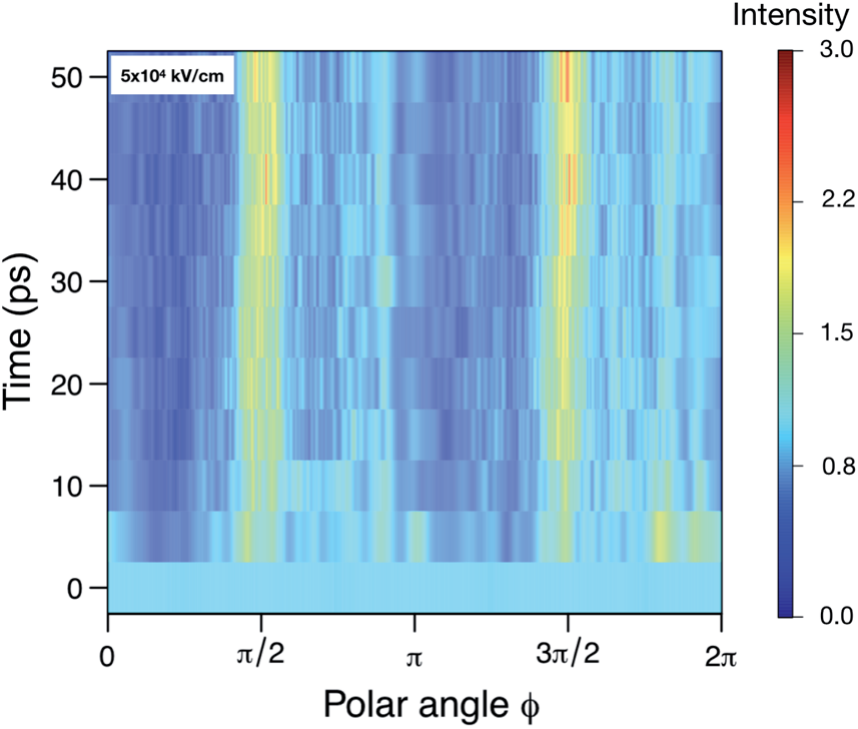
The relative scattered intensity as a function of angle on the detector and time, at a resolution corresponding to 5 Å. The figure shows that the unfolding of the protein is detectable in the anisotropy of the diffracted signal (see ESI Fig. S4,† for diffraction patterns of a folded and unfolded protein). Shown are the simulations at 5 × 10^4^ kV cm^−1^. Please refer to Fig. S5–S7 in the ESI† for plots for the other fields and resolutions.

In contrast to SPI, in SAXS, a pattern averaged over many individual protein samples are recorded in the same diffraction image. We try to estimate the resolution achievable using SAXS by examining the angular intensity distribution within three resolution rings (corresponding to 5 Å, 10 Å and 15 Å) as a function of time. Fig. 4 illustrates this for the 5 Å case. For a sphere-like molecule, the diffraction pattern is fairly isotropic. However, if unfolding occurs under the influence of an electric field, causing the molecule to stretch, the diffraction pattern will accumulate higher intensities at certain angles. For this reason, we expect the distribution to become increasingly angle-dependent as unfolding happens. Therefore, we averaged the 100 analogous diffraction patterns and evaluated the angle-dependency of the mean pixel intensities within the aforementioned rings. For each time point, this yielded three separate functions *I*_*R*_(*θ*) (normalized mean intensity of the elastically scattered photons in each pixel), where the resolution *R* = 5, 10, 15, showing how intensity changes as we trace around the rings. Combining these with the corresponding functions obtained at the remaining time points allowed us to establish their time-dependence, *I*_*R*_(*θ*,*t*). From Fig. 3 we can see that the at a highest resolution we have looked at, 5 Å, the scattered signal will be up to three times higher as the angles corresponding to the unfolding. At lower resolution, the difference in signal is even larger, up to an order of magnitude for 15 Å (see Fig. S5–S7 in the ESI†).

A potential way to detect unfolding induced by electric fields is by using gas phase Förster Resonance Energy Transfer (FRET). FRET is a technique that can measure the distances between two chromophores with high spatial and temporal resolution, and using this technique would have the advantage that we would not need to employ an XFEL or a synchrotron source, but the testing could instead be done in a conventional MS laboratory. Even if gas-phase FRET in theory seems like a good way to detect the unfolding caused by the electric field, there are many experimental challenges that need to be solved before it is actually feasible. The majority of the FRET work done so far was on samples in solution,^47^ however, the potential benefits from combining gas phase FRET with controlled unfolding would be a motivation to develop gas-phase FRET.

To detect unfolding using FRET, it is necessary to find relevant positions where to connect fluorophores to the carbon chain of the protein. We have investigated the distances between all α-carbons in the molecules as a function of time, see Fig. S8 in the ESI.† Analyzing the distances between all pairs of residues over time reveals which regions separate furthest. This analysis shows that this occurs between the tail (residues 65–74) and residue regions 1–7, 14–16, and 32–38 respectively (Fig. S8 in the ESI†). These distances represent the distance between β strand V and I and II respectively, compare to Fig. 1. For FRET analysis of unfolding pathways, these are potential candidates for adding chromophores to the sequence.

## 4 Discussion and conclusions

A reasonable question one can ask is whether the high electric fields discussed in this study can be reached and maintained for the required time to unfold in reality. One of the main concerns, if the field is generated between two electrodes, is electric breakdown, which depends on the quality of both the vacuum and the electrodes. In high vacuum, fields comparable to the ones considered herein have been achieved without breakdown using constant voltages.^48^ Shorter pulses, like on the picosecond-timescales we explore, allow for higher fields than constant voltages,^49^ suggesting that the field requirements, while challenging, can be met. For the four field strengths that we have simulated, we can identify three different behaviors. In the lowest field, the protein does not seem to unfold in a reproducible manner. The states that the protein visits are well-defined (Fig. 2), but the time at which each protein simulation visits a specific state varies a lot, which is reflected in the RMSD (Fig. 1d). Our simulations are 50 ps long, and it is possible that the protein would end up in a well-defined state if the simulations were extended, but that would mean that only the initial and final state are possible to be identified experimentally.

Exposed to the strongest electric field, ubiquitin unfolds along a similar pathway as has been seen in so-called force-induced, or mechanical, unfolding.^22^ MD simulations of the unfolding of ubiquitin by pulling apart the carbon chain has been presented in the literature,^50,51^ and the breaking of the bond between the β-strains follow the order CBD. This is the same dominating order we observe in our simulations using the strongest field. For the lower fields, however, the order changes to CDB, same as has been reported in studies of thermally-induced unfolding of ubiquitin.

We further analyzed the time dependent diffracted signal from the unfolded protein, in order to show that the unfolding should theoretically be possible to study using gas phase SAXS measurements. Our simulations were done on native ubiquitin, and it is possible that adding chromophores (for detection using FRET) to the amino acid chain affects the unfolding. Considering the local amino acid sequence, Daly *et al.* used residue 35 and 73 as mutation sites in a gas phase FRET study.^52^ According to our findings, these positions would also be suitable for detecting unfolding caused by an electric field, which is encouraging, since it is experimentally feasible to place chromophores at these positions. In their study, Daly *et al.* used the chromophores rhodamine 575-C_5_-maleimide as donor and QSY7-C_5_-maleimide as acceptor, which seems to be a reasonable choice to monitor unfolding induced by electric fields. The effect that strong electric fields would have on the efficiency of chromophores as well as unfolding dynamics needs further investigation – but nevertheless, gas phase FRET is potentially a suitable way to detect the induced unfolding.

We acknowledge that the study presented here is based on simulations with classical non-polarizable force fields, and that this only can give qualitative trends. Based on our comparison with first principle calculations of the dipole induced by the field, it is reasonable to believe that the required fields to achieve unfolding are lower than the ones emerging from the MD simulations. Since the required field strengths will be lower than those calculated, it may be less challenging to execute the corresponding experiments. We have used ubiquitin, which is a single-domain protein, as our model system, in part because it has been extensively studied in the gas phase, but also due to its manageable size. We speculate, however, that other proteins might be more amenable to field-induced unfolding. For example, it has been shown that weaker electric fields can perturb the structures of tubulin dimers^15^ and polyalanines.^16^ Future investigations could also attempt domain-wise unfolding in multi-domain proteins or even subunit unfolding in multimeric proteins, which could be easier to detect experimentally due to the larger size of the structural units compared to the β-strands monitored herein.

## Conflicts of interest

There are no conflicts to declare.

## Supplementary Material

SC-012-D0SC06008A-s001
