# Supplementary material for: Reproducibility in the unfolding process of protein induced by an external electric field
Source: Chem Sci. 2020 Dec 26;12(6):2030–8. doi: 10.1039/d0sc06008a (PMC8179335; doi:10.1039/d0sc06008a)
Supplement: SC-012-D0SC06008A-s001 [file SC-012-D0SC06008A-s001.pdf]

Electronic Supplementary Material (ESI) for Chemical Science.
This journal is © The Royal Society of Chemistry 2020

Reproducibility in the unfolding process of protein induced by an external electric field - Supplementary Information

Anna Sinelnikova,[†] Thomas Mandl,[†,¶] Christofer Östlin,[†] Oscar Grånäs,[†] Maxim N. Brodmerkel,[‡] Erik G. Marklund,[‡] and Carl Caleman[*,†,§]

†*Department of Physics and Astronomy, Uppsala University, Box 516, SE-751 20 Uppsala, Sweden*

‡*Department of Chemistry – BMC, Uppsala University, Box 576, SE-751 23 Uppsala, Sweden*

¶*University of Applied Sciences Technikum Wien, Höchstädtplatz 6, A-1200 Wien, Austria*

§*Center for Free-Electron Laser Science, DESY, Notkestrasse 85, DE-22607 Hamburg, Germany*

E-mail: carl.caleman@physics.uu.se

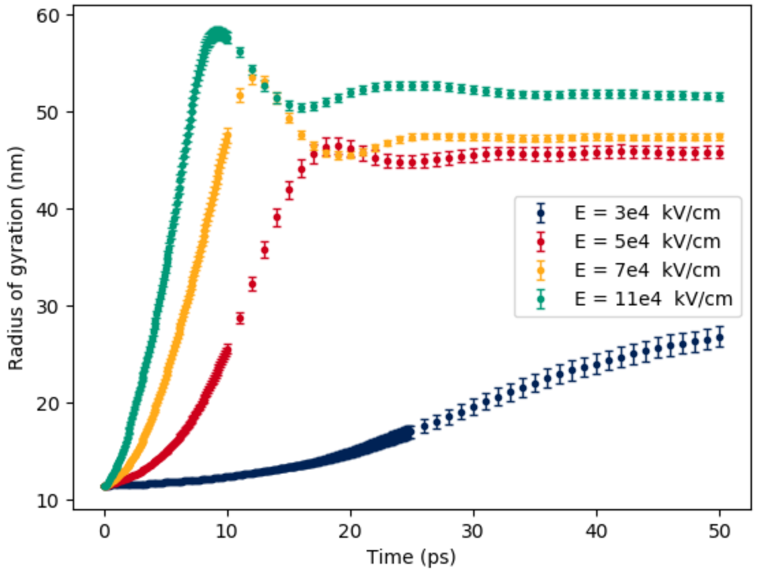

Figure S1: Average radius of gyration, and standard deviation of the average value for the simulations in the four different electric fields. Each line represents a set of 100 independent simulations, and the radius of gyration is calculated at each time step comparing the 100 structures structures at this specific time in the simulations.

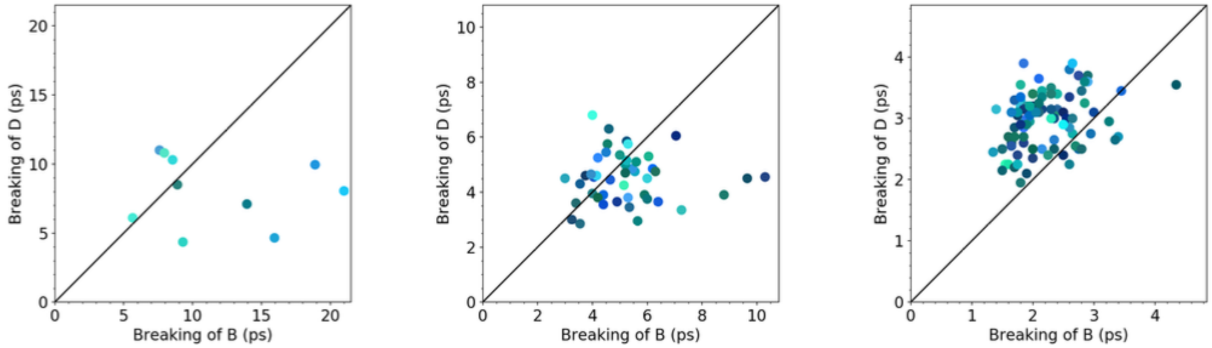

(a) $E = 5 \cdot 10^4$ kV/cm. **(b)** $E = 7 \cdot 10^4$ kV/cm. **(c)** $E = 11 \cdot 10^4$ kV/cm.

Figure S2: The time points for the breaking of B and D at three fields strengths. With increasing E-field the probability for B breaking before D increases. At higher fields B is broken before D. Each point corresponds to a single simulation.

Table 1: Dipole vs. field for folded and unfolded TRP cage, units in Debye.

E (kV/cm)	Folded	Unfolded
No field	29	28
$3 \cdot 10^4$	53	71
$5 \cdot 10^4$	83	122
$7 \cdot 10^4$	128	194
$11 \cdot 10^4$	251	352

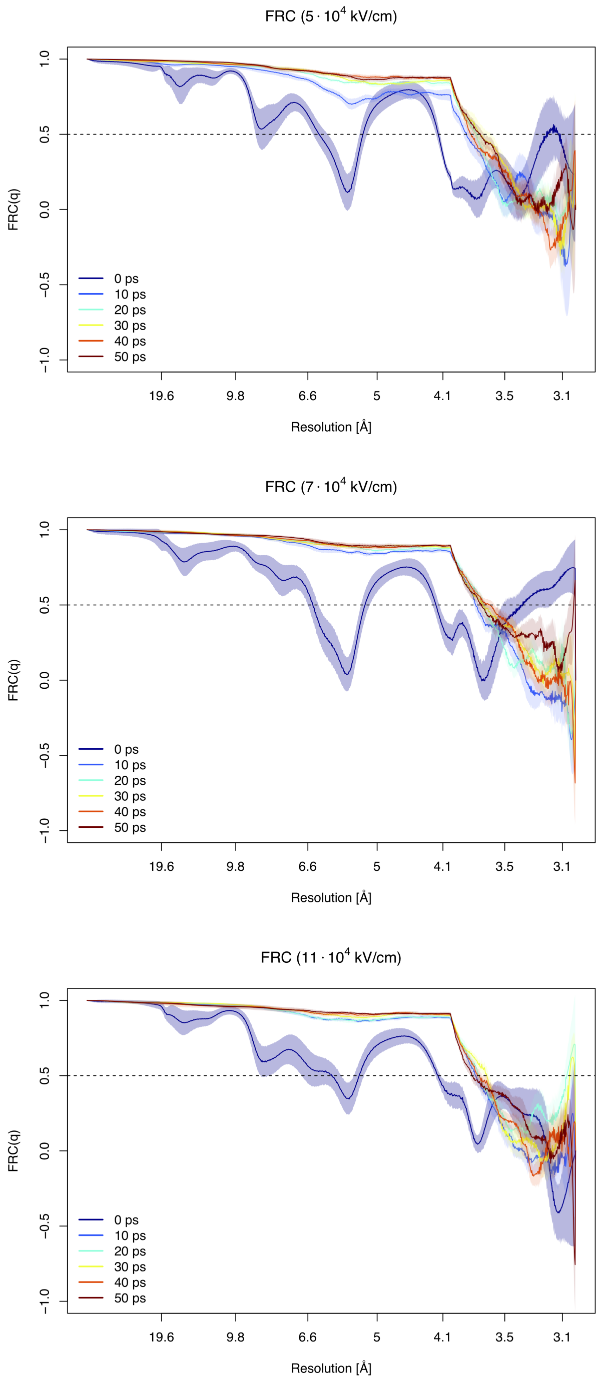

Figure S3: Estimated resolution limit, based on Fourier ring correlation (FRC) for different time points in the simulation. Field strengths above $E = 3 \cdot 10^4$ kV/cm yield in an achievable resolution of the unfolding process around 4 Å.

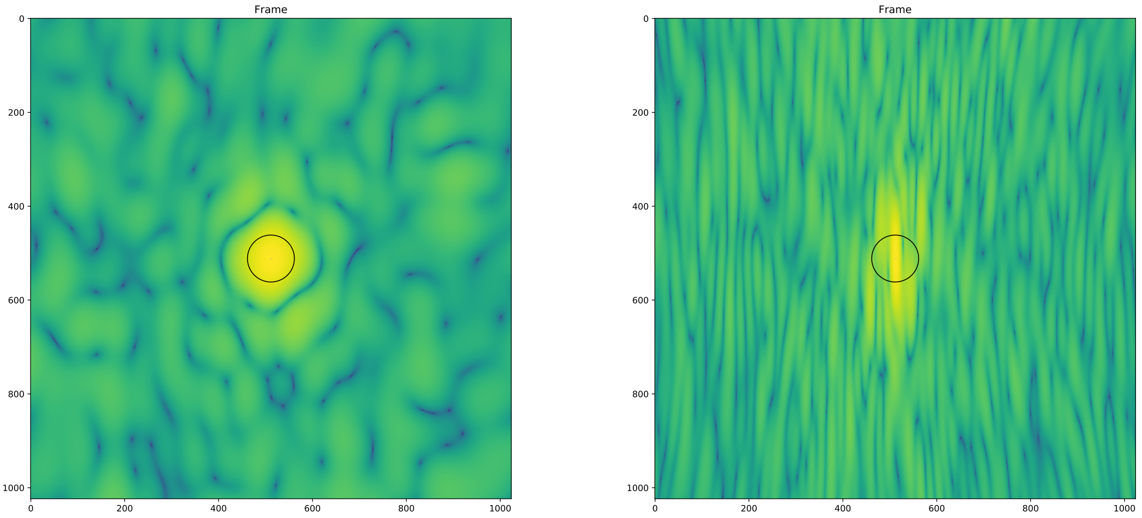

Figure S4: Simulated diffraction patterns from the molecular dynamics trajectories of the $E = 3 \cdot 10^4$ kV/cm simulation. The diffracted image of the initial, folded structure does not have any expressed angular dependence (left panel), whereas the the diffracted image of the final, unfolded (right panel) has a strong angular dependence. The ring in the patterns shows the 15 Å resolution shell.

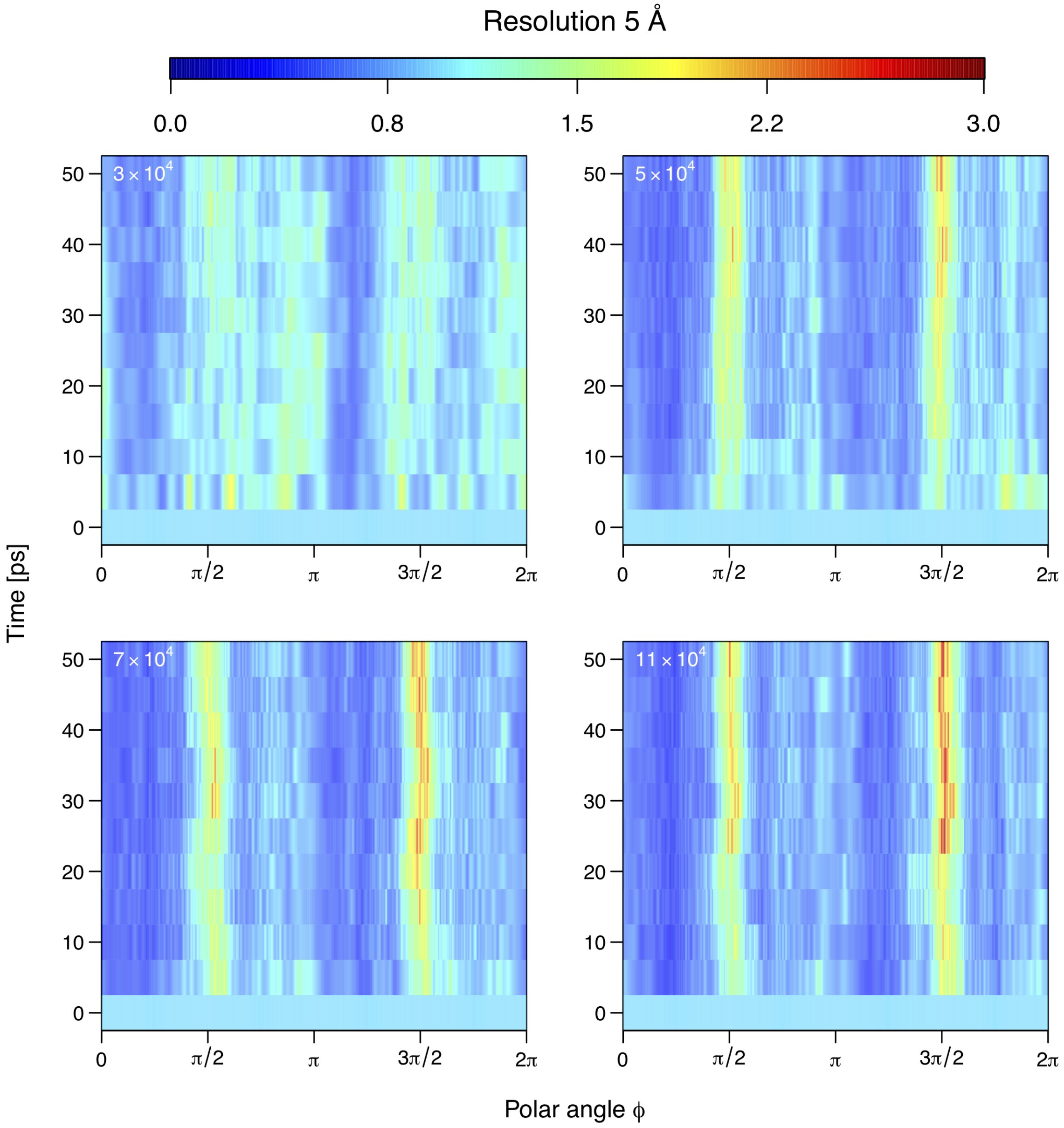

Figure S5: The relative scattered intensity as a function of angle on the detector and time, at a resolution of 5 Å for all studied electric field strengths.

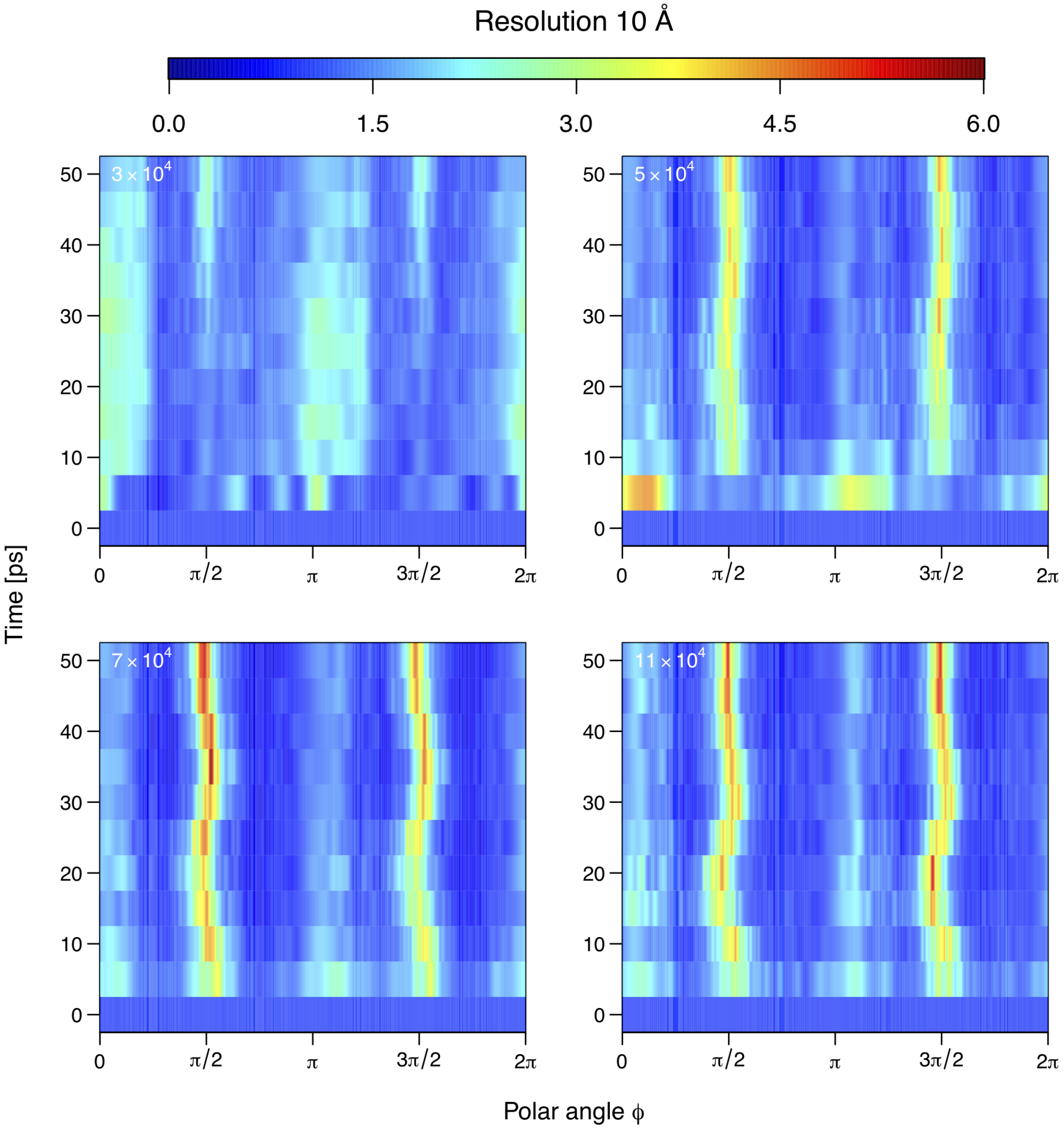

Figure S6: The relative scattered intensity as a function of angle on the detector and time, at a resolution of 10 Å for all studied electric field strengths.

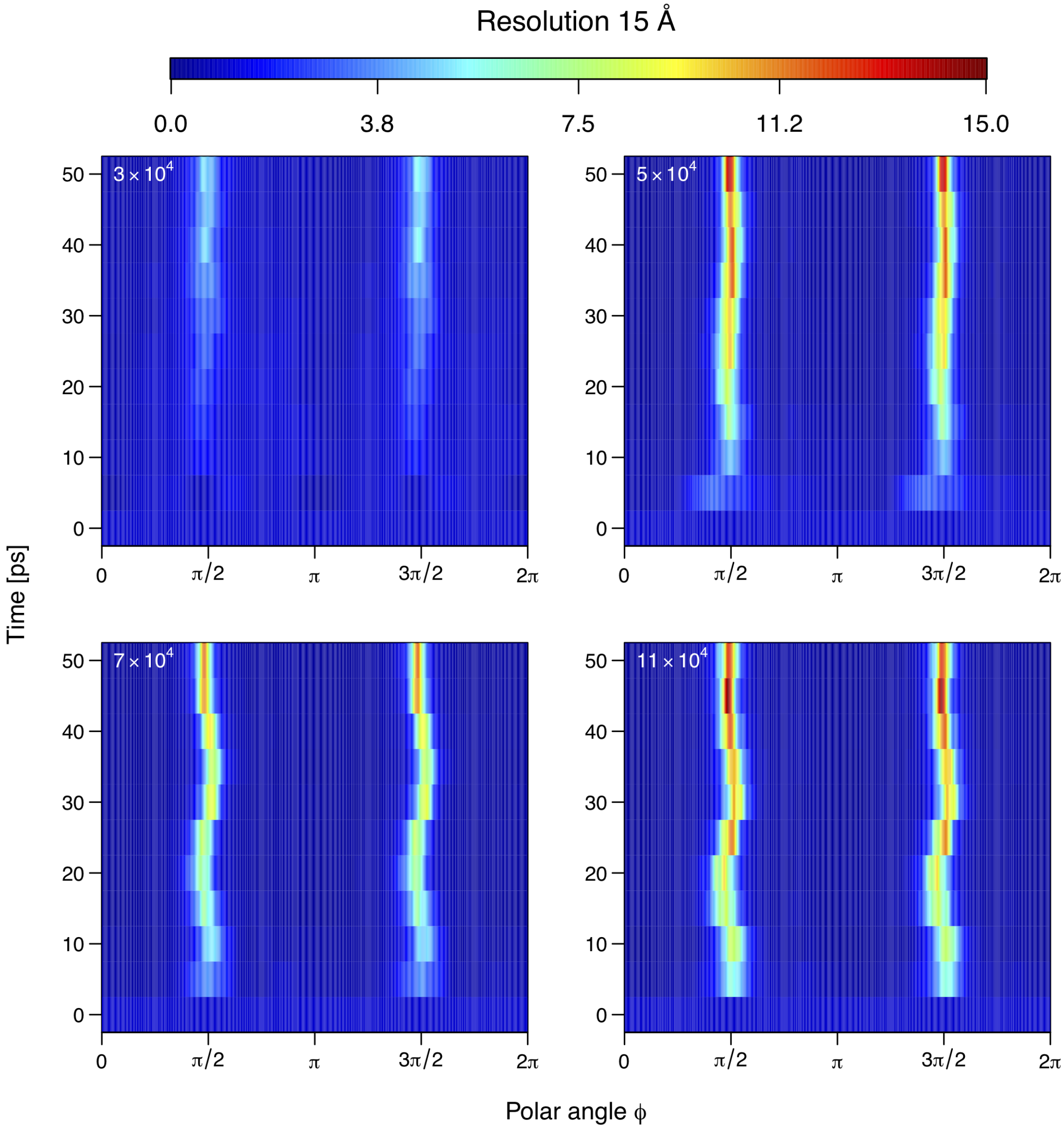

Figure S7: The relative scattered intensity as a function of angle on the detector and time, at a resolution of 15 Å for all studied electric field strengths.

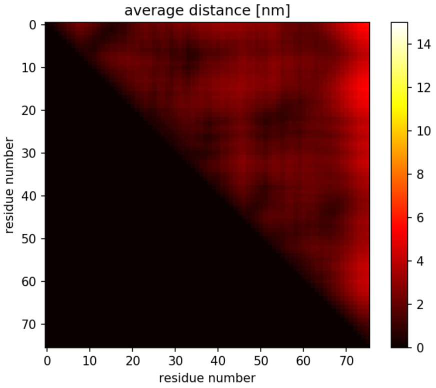

(a) $t = 0 - 10$ ps, $E = 5 \cdot 10^4$ kV/cm.

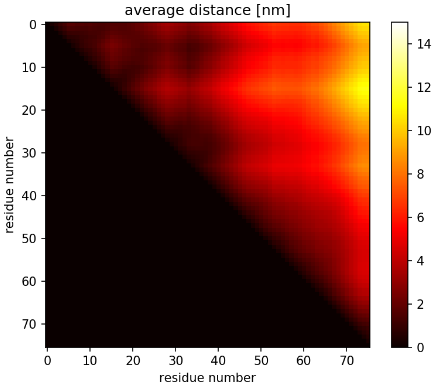

(b) $t = 0 - 10$ ps, $E = 11 \cdot 10^4$ kV/cm.

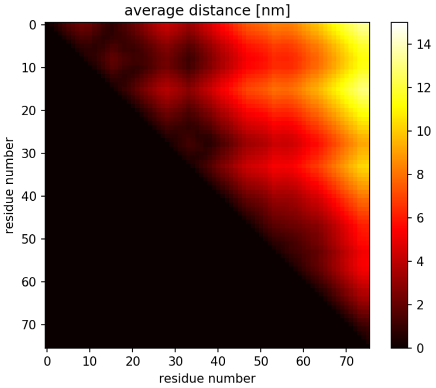

(c) $t = 10 - 20$ ps, $E = 5 \cdot 10^4$ kV/cm.

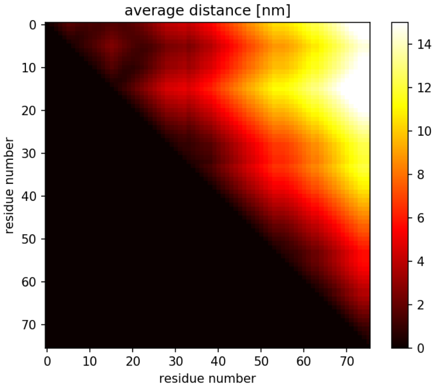

(d) $t = 10 - 20$ ps, $E = 11 \cdot 10^4$ kV/cm.

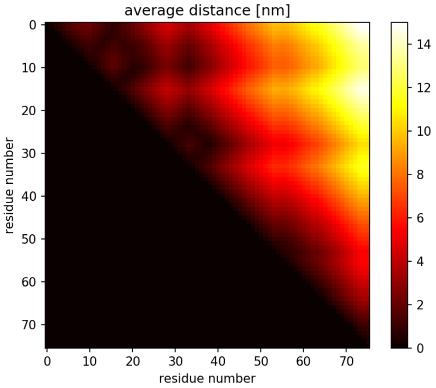

(e) $t = 20 - 50$ ps, $E = 5 \cdot 10^4$ kV/cm.

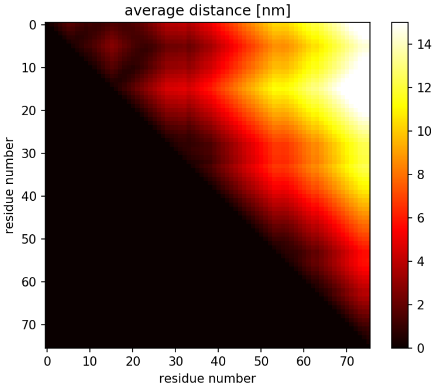

(f) $t = 20 - 50$ ps, $E = 11 \cdot 10^4$ kV/cm.

Figure S8: Average distance between all residues for the ubiquitin at E = $5 \cdot 10^4$ and E = $11 \cdot 10^4$ kV/cm. **a.** and **b.** Average for the first 10 ps of the simulations. **c.** and **d.** Average between 10 and 20 ps of the simulations. **e.** and **f.** The largest changes in the distances are between residues 1-7 and 65-74, 14-16 and 65-74, and 32-38 and 65-74, which should be good candidates for chromophores to detect unfolding using FRET. The color of a single pixel represents the average distance between the α-carbons of a single pair of residues in the indicated time slice. Darker areas indicate lower distances, brighter colors larger distances (only upper triangular matrix shown).
